# Mental health problems in relation to eating behavior patterns, nutrient intakes and health related quality of life among Iranian female adolescents

**DOI:** 10.1371/journal.pone.0195669

**Published:** 2018-04-27

**Authors:** Mahdieh Abbasalizad Farhangi, Parvin Dehghan, Leila Jahangiry

**Affiliations:** 1 Nutrition Research Center, Tabriz University of Medical Sciences, Tabriz, Iran; 2 Drug Applied Research Center, Department of Nutrition and Biochemistry, Tabriz University of Medical Sciences, Tabriz, Iran; 3 Tabriz Health Services Management Research Center, Health Education and Health Promotion Department, Tabriz University of Medical Sciences, Tabriz, Iran; TNO, NETHERLANDS

## Abstract

**Aims:**

To identify the association between mental health problems, eating behavior patterns, nutrient intakes and health related quality of life (HRQoL) among Iranian female adolescents.

**Materials and methods:**

The current cross-sectional study conducted among three high-schools randomly selected from 10-day-public high schools in the selected sub-county from Tabriz city-Iran between December 2015 through March 2016. Participants were a sample of 107 adolescent girls aged 15–17 years old. Anthropometric parameters were measured and assessments of HRQoL, mental health problems and eating behavioral patterns were performed by Short Form 36 (SF-36), Strengths and Difficulties Questionnaires (SDQ) and Eating Behavioral Pattern Questionnaire (EBPQ) respectively. Dietary intake was assessed using a semi-quantitative Food-Frequency Questionnaire (FFQ) adapted for the Iranian society. Quality of life was measured with HRQoL questionnaire. Quantitative analysis using independent sample *t* test was performed for comparison of continuous variables between two subgroups (unlikely, possible/probable) of each category of mental health problem. Multiple logistic regression was used to measure the potential predictors (e.g. eating patterns and HRQoL) of mental health problems in two models of crude and adjusted for age and body mass index (BMI). P values less than 0.05 were considered as statistically significant.

**Results:**

Indicators of conduct problems and hyperactivity disorders were the most prevalent mental health problems among female adolescents (25.2% and 18.6% respectively). The prevalence of hyperactivity disorders among female adolescents was 35.5%. Female adolescents in high scores of ‘snacking and convenience’, ‘planning ahead’ and ‘meal skipping’ eating patterns were more likely to have indicators of emotional disorders (P < 0.05). Also, being in the high tertile of ‘low fat eating’ pattern made adolescents less likely to have hyperactivity disorders (P < 0.05). Moreover, according to our finding, high scores of vitality and mental health components of HRQoL were associated with reduced likelihood of emotional disorders, conduct disorders and hyperactivity disorders. No significant difference in terms of BMI and nutrient intakes in different categories of mental health problems according to SDQ scoring was identified.

**Conclusions:**

In the current study unhealthy eating patterns including ‘snacking and convenience’, ‘planning ahead’ and ‘meal skipping’ eating patterns were positive predictors of “emotional disorders” while “low fat” eating pattern was negative predictor of hyperactivity disorders.

## Introduction

Mental health problems comprise of a broad range of mental disorders including anxiety disorders, bipolar disorders, depression and eating disorders are generally characterized by some combination of abnormal thoughts, emotions, behavior and relationships with others [1]. Mental health problems, as common public health issues among adolescents, have a significant adverse impact not only on adolescents but also on their parents and families particularly in relation to health related quality of life (HRQoL) [2, 3]. Health-related quality of life (HRQoL) is a multi-dimensional concept that includes domains related to physical, mental, emotional, and social functioning. It goes beyond direct measures of population health, life expectancy, and causes of death, and focuses on the impact of the health status on health related quality of life [4, 5]. Adolescence, as a transitional period between childhood and adulthood, is associated with dramatic change and development of the physical, emotional and cognitive function [6]. Unhealthy eating patterns including meal skipping, snacking sugar containing drinks and sweets, eating away from home, fast food consumption and high intakes of simple sugars and saturated fats are common among adolescents [2, 6]. Adequate nutrition and healthy food choices are known to be an important factor in the development of brain and prevention of cognitive disorders; unhealthy eating behaviors are important determinants of mental health problems including hyperactivity disorder, depression and conductive problems in adolescents [7, 8].

Numerous studies have suggested an association between unhealthy eating habits and mental health problems in adolescents. In the study by Oellingrath et al [2] significant associations between unhealthy eating patterns including “junk/ convenient” and “snacking” eating patterns with hyperactivity/ convenient disorder and conduct/ oppositional disorders among Norwegian adolescents were identified. In a population-based cohort of adolescents by Howard et al [9] high scores of western dietary pattern include high intakes of total and saturated fat, refined grains and sugars, processed meat and sodium, and lower intakes of fruits, vegetables, omega-3 fatty acids and fiber were associated with attention deficit hyperactivity disorders (ADHD) diagnosis (odds ratio = 2.21).

In several other studies, dietary patterns and poor health behaviors were associated with depressive disorders and anxiety [9, 10]. In the study by Jacka et al [11] improvements in diet quality and healthy diets were associated with improvements in mental health in adolescents. Breakfast skipping and high consumption of sugar-containing soft drinks and sweets were associated with various mental health problems in three studies [8, 12, 13]. These studies further highlight the potential association between unhealthy nutritional habits and mental health problems in adolescents.

There are several potential biologic explanations for the relationship between eating patterns and mental health in adolescents. First of all, the poor dietary quality that leads to lack of nutrient-dense foods could be associated with mental problems; for example a diet low in folate, zinc, magnesium and long chain omega-3 fatty acids is associated with depressive and anxiety disorders [14, 15] Second possible explanation is the direct effect of diet on the pathological pathways and biologic mechanisms of the mental disorders including the direct effect of diet on inflammatory parameters, immune system markers and biomarkers of oxidative stress responsible for mental issues like depression or anxiety. Studies have revealed that poor diet is directly associated with higher inflammation and oxidative stress which finally leads to mental disorders [16, 17]. Moreover, diets high in simple sugars and saturated fats could affect proteins involved in the brain development like brain derived neurotropic factor (BDNF) [18].

Accordingly, several studies have also revealed the association between HRQoL and diet; adherence to healthy diets is associated with significant improvements in general physical and psychological health, longevity and lower levels of obesity [19, 20]. In a population-based study conducted by Plaisted et al adherence to fruits and vegetable diet or combination diet including fruits, vegetables and low fat dairy products for three weeks were associated with improved subscales of HRQoL; these improvements were 5% and 5.9% from baseline for these two diets respectively [21]. Adherence to healthy diets like dietary approach to stop hypertension (DASH) or Mediterranean dietary patterns without feeling of deprivation and hunger could help people to maintain their quality of life [21]. Adolescents with good adherence to the Mediterranean dietary patterns scored significantly higher in total HRQoL and in the physical well-being, psychological well-being, parents and autonomy, and peers and social support [22].

Recently, studies about dietary patterns have attracted potential attention since dietary patterns provide an overview of diet and facilitate exploration of overall diet and diet-disease relationship that are not revealed when single nutrients or food items are studied [23]. In the current study, mental health problems were defined as three aspects of mental issues including conduct/oppositional disorders, hyperactivity/ inattention disorders and anxiety/ depressive disorders measured by a self-completed strengths and difficulties questionnaire (SDQ) [24].

Female adolescents are more prone to develop internalizing mental health problems including emotional problems, depression and anxiety [25]. This is because female adolescents use more internalizing mechanisms to face problems [26]. Child and adolescent’s mental health problems are classified as internalizing and externalizing emotional and behavioral problems. Externalizing problems are consisted of abnormal mental behaviors including problems with controlling unwanted behavior, attention and cognitive processing and include conduct disorder (CD), ADHD, and oppositional defiant disorder (ODD). On the other hand, internalizing problems are characterized by an inability to control negative emotions, such as depression, rumination, loneliness, anxiety and sadness [27]. Externalizing problems are more common in boys, while internalizing problems are more prevalent in girls [26]. Unhealthy eating patterns are most likely to develop internalizing rather than externalizing problems and are more common among girls [28]. Teenage girls eat less healthy foods than other population groups; lower self-esteem, more body image dissatisfaction and distortion and thin ideals in the modern society also trigger the unhealthy eating patterns and related mental issues in females [26, 29].

Mental health problems refer to a wide range of disorders including anxiety disorders, bipolar disorders, depression and eating disorders [30]. These mental health problems are more common in women than in men and gender bias in the prevalence of mental disorders particularly occur in the cases of common mental disorders like depression, anxiety and somatic complaints [30]. Females face different roles in society that render them at greater risk of experiencing mental disorders than others in the community. Studies showed that the prevalence of some mental disorders, like depression and anxieties and psychological distress are higher among women compared with men [31, 32].

The prevalence of mental health issues in Iran also is in accordance with corresponding values in other societies; in 2004, according to the study by Noorbala et al, 21% of the total population including 25.9% of women and 14.6% of men suffered from mental disorders [33]. In 2008, these values had been increased to 36% in Tehran with the rate of 2 to 1 in women than men [34].

Moreover students encounter with higher stress than others because of greater material expectations, greater pressure on academic institutions and staff, change in environment, and expansion of student numbers. This stress with serious consequences leads to the development of mental problems [35]. Students’ material expectations include need to supportive educational environment, constructive criticism, organized lectures, supportive materials, clear expectations from different examinations, homework, respect for student beliefs, desire to discuss about student educational concerns, accessible class-related meetings and conferences [36]. Other sources of stress include financial problems, domestic responsibilities, holding a job while in school, and a heavy academic responsibilities as established by Phinney et al [37]. Overall, the students’ stressors could be classified as three classes of relationships, personal factors include change in sleeping habits, new responsibilities, having health problems and poor eating habits and academic stressors include increase class workload, lower grades, many hours of studies, language difficulties and procrastination and also environmental factors include lack of vacation of breaks, bad living conditions, computer problems, divorce between parents, future worries and etc [38, 39]. Therefore it seems necessary to evaluate the relationship between eating patterns and mental health issues among teenage student girls.

In the current study we aimed to: (1) evaluate the mental health problems in the female adolescents using SDQ questionnaire and (2) investigate the association of mental health problems with eating behavior patterns and HRQoL among female adolescents in Tabriz- Iran.

## Materials and methods

### Subjects

The research protocol of the current study has been approved by the ethics committee of Tabriz University of Medical Sciences. The study was conducted among a sample of one hundred seven female adolescents aged between 15–17 years randomly selected from three high schools of Tabriz in northwest of Iran between December 2015 through March 2016. Three high schools were randomly selected from 10 day-public high schools in the selected sub-county. Proportionate to size sampling was used to sample 107 teenagers. At the school level random sampling was used to select children from each school. Participants were all living in Tabriz city, were from middle-class families and all had Iranian eating habits. Typical Iranian main dishes are combinations of rice with meat, vegetables, and nuts. Fresh green herbs are frequently used, along with fruits such as plums, pomegranate, quince, prunes, apricots, and raisins. Characteristic Iranian flavorings such as saffron, dried lime, cinnamon, and parsley are mixed and used in some special dishes. Moreover, the Iranian eating habits (in terms of meal sizes and timing) would be generally quiet Mediterranean- A standard, simple breakfast, a large lunch and a light dinner- with desserts or fruit before bed [40].

The parents or legal guardians of all participants signed a written informed consent approved by the Institutional Review Board of Tabriz University of Medical Sciences. All investigations have been conducted according to the principles expressed in the Declaration of Helsinki. All of the study measurements including anthropometric assessments and questionnaires administrations were performed by research team including two researchers trained about study measurements.

### Anthropometric assessments

Weight was measured by calibrated Seca scale (Itin Scale Co., Inc. Germany) with the precision of 0.1 kg and height to the nearest 0.1 cm with a wall scale while subjects wearing light clothes and no shoes. The measurements were performed one time and the students were informed about the results of their anthropometric measurements in written forms. BMI was calculated as weight (kg) divided by height (m) ^2^. Obesity was defined as BMI ≥ 30 kg/m^2^ [41].

### Measurement of health-related quality of life (HRQoL)

Health related quality of life was measured using the Short Form 36 (SF-36) questionnaire first developed by Ware et al [42] designed to provide an instrument for the self-evaluation of health related quality of life. The questionnaire was validated in a large population based study among Iranian population aged over 15 by Montazeri et al [43] for using in Iranian population. The questionnaire includes 36 items measuring eight dimensions of life quality: Physical Functioning (PF); Role Physical (RP), which refers to role limitations due to physical difficulties; Bodily Pain (BP); General Health (GH); Vitality (VT); Social Functioning (SF); Role Emotional (RE), which refers to role limitations due to emotional difficulties, and Mental Health (MH). The SF-36 has eight scaled scores; the scores are weighted sums of the questions in each section. Scores range from 0 to 100. Lower scores denote more disability and poor HRQoL while higher scores denote less disability and better HRQoL [43]. The instrument achieved a *Cronbach’s α* of 0.76 in the current study.

### Prediction of mental health problems

Prediction of mental health problems was performed by the Strengths and Difficulties Questionnaires (SDQ), a brief screening questionnaire for psychological problems of children and adolescents. The questionnaire includes 25 items. Each of the 25 items is scored on a three point Likert type scale ranging from 0 to 2. The items are divided into five subscales with five items each resulting in scores of emotional disorders, conduct problems, hyperactive behavior, peer relationships and pro-social behaviors. The combinations of scores for each sub scales are analyzed using an algorithm defined by Goodman et al [44]. This algorithm make separate predictions for indications of emotional problems, conduct disorders and hyperactivity disorders, peer problems and pro-social behaviors. The algorithm predicts mental health as being “probable” (high risk), “possible” (borderline) or “unlikely” (normal). In the current study, we combined the “possible” and “probable” categories into “possible/probable”, and all comparisons were made between this category and the category “unlikely”. This combination made the groups. The questionnaire may be completed by parents or teachers of children aged 4–10 years or as a self-report questionnaire completed by children of 10–17 year old themselves [45]. In the current study we used a self-completed SDQ completed by students. The questionnaire was validated by Ghanizadeh et al [24] for use in Iranian children and teenagers aged 3 to 18 years old. The questionnaire achieved the *Cronbach’s* α of 0.71 in the current study.

### Measurement of eating behavioral pattern

Eating behavioral patterns were measured by eating behavioral pattern questionnaire (EBPQ). The questionnaire was firstly developed in Vanderbit University to evaluate dietary fat intake among African- American women [46]. The questionnaire includes 51 self-reported items on healthy and unhealthy eating behaviors. Every item was rated in a 5 point Likert scale ranging from strongly disagrees to strongly agree. Six eating behavior patterns were assessed in the EBPQ as follows: low fat eating (questions about the amount of fat in the diet and choosing low fat foods), snacking and convenience (questions about frequent use of cookies, high sugar bars and other sweet snacks), emotional eating (questions about the effect of emotional change on eating patterns), planning ahead (questions about eating out of home and frequent eating of fast foods in restaurants), meal skipping (questions about skipping the main food meals) and cultural/ life style behavior (questions about the role of food in family and community and the role of cultural impositions on food choices). Previous studies in Iran demonstrated the initial validity, reliability and feasibility of the Iranian version of EBPQ for use in young females establishing the *Cronbach’s α* and intra-class correlation coefficient (ICC) ranging between 0.55 to 0.78 and 0.67 to 0.89 respectively [47]. However its reliability was assessed again and Cronbach’s *α* of 0.78 was established in the current study.

### Dietary intake

Dietary intake was assessed using a semi-quantitative food-frequency questionnaire (FFQ) of 168 food items adapted to the Iranian society [48]. The FFQ included with specified serving sizes commonly consumed by Iranians. Participants reported their average frequency intake of each food item in terms of the number of specified serving sizes consumed per day/ week/ month/ year, or never. The reported frequency of consumed foods and portion sizes for each food item were converted to a daily intake.

### Statistics

Statistical analysis was performed with Statistical Package for Social Science (SPSS 18 for windows, SPSS Inc^®^ headquarter, Chicago, USA). Normality of data was analyzed by Kolmogorov-Smirnov test. Chi square and independent sample *t* test were performed for comparison of discrete and continuous variables between two subgroups (unlikely, possible/probable) of each category of mental health problem respectively. Multiple logistic regression analysis was used to examine the association between mental health problems as dependent variable and eating patterns and health related quality of life as independent variables. Adjusted odds ratio (OR) and 95% confidence intervals (CI) were calculated for emotional, conduct, hyperactivity, peer and prosocial problems. The potential confounding variables entered in the multiple logistic regression analysis were age and body mass index. Two models of logistic regression results have been presented. A crude OR and an adjusted OR which is controlled for confounding variables. P values less than 0.05 were regarded as statistically significant.

## Results

The prevalence of mental health problems according to SDQ scores are presented in Table 1. Indicators of conduct problems, hyperactivity disorders and emotional disorders were the most prevalent mental health problems among female adolescents; while the indicators of prosocial problems and peer problems were reported less frequently. In comparison of BMI and nutrient intakes in different categories of mental health problems according to SDQ scoring, no significant difference was reported (Table 2). Multiple logistic regression analysis evaluating the association between mental health problems and eating patterns (Table 3) showed that female adolescents in high scores of “snacking and convenience”, “planning ahead” and “meal skipping” eating patterns were more likely to have indicators of “emotional disorders” than those in lower scores of these eating patterns even after adjusting for confounding effects of age and BMI. Moreover, adolescents in high scores of “low fat” eating pattern were less likely to have hyperactivity disorders. Adolescents in high scores of “planning ahead” eating pattern were more likely to have increased likelihood of peer problems and pro-social problems. High scores of “meal skipping” eating pattern were associated with increased likelihood of emotional disorders and hyperactivity disorders.

**Table 1:** The prevalence of mental health problems according to the SDQ symptom scores in female adolescents.

Mental health problems	Category	N	%
Indicators of emotional disorders	Disorder unlikely	77	70.6
	Disorder possibly	15	14.0
	Disorder probable	15	14.0
Indicators of conduct disorders	Disorder unlikely	58	53.2
	Disorder possibly	22	20.6
	Disorder probable	27	25.2
Indicators of hyperactivity disorders	Disorder unlikely	69	63.3
	Disorder possibly	18	16.8
	Disorder probable	20	18.7
Indicators of peer disorders	Disorder unlikely	74	68.0
	Disorder possibly	29	27.1
	Disorder probable	4	3.7
Indicators of prosocial problems	Disorder unlikely	91	83.5
	Disorder possibly	6	5.6
	Disorder probable	10	9.3

**Table 2:** The comparison of BMI and nutritional intakes in different categorizes of mental health problems according to SDQ scores.

Variable	Indicators of emotional disorders		Indicators of conduct disorders		Indicators of hyperactivity disorders		Indicators of peer problems		Indicators of prosocial problems	
(Mean ±SD)	Unlikely	Possible / probable	Unlikely	Possible / probable	Unlikely	Possible / probable	Unlikely	Possible / probable	Unlikely	Possible / probable
**Age (y)**	16.14 ± 0.72	17.38 ±0.62	16.25 ± 0.71	17.14 ±7.72	17.01± 6.63	16.03± 0.67	16.25± 0.72	17.14± 7.88	16.20 ±0.68	19.60 ±14.24
**BMI (kg/m**^**2**^**)**	20.28 ± 2.70	20.16 ±2.77	20.35 ±2.88	19.92 ±2.56	20.13 ± 2.77	20.18 ±2.60	19.98±2.73	20.19 ±2.69	20.09± 2.61	20.11 ± 3.24
**Energy (kcal/d)**	1758.37 ± 681.84	1900.8± 668.05	1777.51± 736.10	1784.34± 631.70	1775.24± 676.35	1845.24 ± 674.19	1775.04± 683.30	1806.51± 678.90	1777.83± 677.95	1837.58 ± 679.34
**Protein (g/d)**	2.21 ± 0.84	1.97 ± 0.44	64.53 ±31.38	64.86 ±26.01	65.82± 29.64	64.55± 25.97	62.95 ± 29.29	66.95 ±27.61	63.91 ±28.28	69.83 ± 28.86
**CHO (g/d)**	237.21± 96.77	267.83 ± 102.82	246.84± 102.06	244.84 ±101.03	240.20± 94.50	263.52± 112.11	240.25 ± 94.46	253.49 ± 107.52	244.03± 95.06	261.11 ± 129.21
**Fat (g/d)**	62.40 ±2.12	66.62 ± 3.50	62.43± 36.35	62.57 ±32.73	64.05± 35.72	62.06 ±30.72	65.03 ±37.20	61.25 ±31.69	63.45 ± 35.99	59.98 ± 21.89
**SFA (g/d)**	19.07 ±9.80	23.27 ± 19.03	19.53 ±11.17	21.48 ±18.10	21.74± 17.11	18.99± 8.33	20.85± 17.88	20.35± 10.49	21.01 ±15.60	18.03 ± 7.61
**PUFA(g/d)**	13.76 ±10.09	12.75 ± 8.46	13.25± 10.04	12.66 ±8.56	13.12 ±9.34	12.43 ±8.57	14.07 ±10.49	11.78 ±7.41	13.06 ±9.45	11.81 ± 7.23
**MUFA(g/d)**	22.56 ±16.57	26.49 ±23.72	21.81 ±15.88	25.70 ±22.91	23.20 ± 16.23	25.60± 26.12	24.13± 18.87	24.14± 21.19	24.31± 21.00	20.77 ± 10.62

BMI, body mass index; CHO, carbohydrate; SFA, short chain fatty acids; PUFA, poly unsaturated fatty acids; MUFA, monounsaturated fatty acids. Statistics were performed by independent sample t-test and no significant difference was observed.

**Table 3:** Association between tertiles of eating patterns scores and mental health problems among adolescents.

Eating patterns	N	Indicators of emotional disorders				Indicators of conduct disorders				Indicators of hyperactivity disorders				Indicators of peer problems				Indicators of prosocial problems			
		Crude		Adjusted †		Crude		Adjusted†		Crude		Adjusted †		Crude		Adjusted †		Crude		Adjusted†	
		OR	95% CI	OR	95% CI	OR	95% CI	OR	95% CI	OR	95% CI	OR	95% CI	OR	95% CI	OR	95% CI	OR	95% CI	OR	95% CI
**Low fat eating pattern**																					
Tertile1	31	1	Ref.	1	Ref.	1	Ref.	1	Ref.	1	Ref.	1	Ref.	1	Ref.	1	Ref.	1	Ref.	1	Ref.
Tertile2	32	0.82	0.19–1.60	0.81	0.28–2.24	0.37	0.13–1.05	0.39	0.14–1.12	**0.36**	**0.13–1.02**	0.35	0.12–1.01	**2.96**	**1.10–7.94**	**2.97**	**1.14–8.07**	0.47	0.14–1.62	0.47	0.13–1.71
Tertile3	27	0.56	0.31–2.27	0.61	0.21–1.80	**0.16**	**0.05–0.52**	**0.18**	**0.05–0.58**	0.41	0.14–1.24	0.37	0.12–1.16	1.88	0.66–5.31	1.93	0.66–5.64	**0.11**	**0.01–0.91**	**0.11**	**0.01–0.91**
**Emotional eating pattern**																					
Tertile1	32	1	Ref.	1	Ref.	1	Ref.	1	Ref.	1	Ref.	1	Ref.	1	Ref.	1	Ref.	1	Ref.	1	Ref.
Tertile2	33	1.21	0.86–7.11	1.21	0.45–3.29	1.13	0.44–2.94	1.11	0.42–2.89	0.35	0.12–1.01	0.93	0.32–2.72	0.85	0.33–2.19	0.84	0.33–2.18	2.07	0.54–7.85	2.12	0.55–8.09
Tertile3	27	2.48	0.45–3.25	2.33	0.79–6.83	0.87	0.32–2.39	0.85	0.31–2.40	0.37	0.12–1.16	3.15	1.06–9.24	0.92	0.34–2.48	0.99	0.36–2.73	0.92	0.18–4.49	0.61	0.11–3.53
**Snacking and convenience**																					
Tertile1	34	1	Ref.	1	Ref.	1	Ref.	1	Ref.	1	Ref.	1	Ref.	1	Ref.	1	Ref.	1	Ref.	1	Ref.
Tertile2	28	1.40	0.51–3.86	1.47	0.52–4.18	0.50	0.18–1.36	0.50	0.18–0.36	0.72	0.24–2.16	0.73	0.24–2.25	0.59	0.23–1.57	0.63	0.24–1.67	0.35	0.06–1.90	0.42	0.07–2.37
Tertile3	32	**2.69**	**0.99–7.28**	**2.88**	**1.03–8.04**	1.57	0.60–4.13	1.57	0.59–4.19	2.22	0.82–6.0	2.36	0.83–6.42	0.88	0.34–2.31	0.94	0.35–2.46	1.4	0.33–3.98	1.37	0.37–5.07
**Planning ahead eating pattern**																					
Tertile1	32	1	Ref.	1	Ref.	1	Ref.	1	Ref.	1	Ref.	1	Ref.	1	Ref.	1	Ref.	1	Ref.	1	Ref.
Tertile2	33	0.95	0.35–2.56	0.95	0.34–2.65	1.77	0.67–4.69	1.90	0.73–4.94	1.72	0.61–4.81	1.72	0.61–4.91	**3.60**	**1.32–9.79**	**3.81**	**1.36–10.6**	**8.25**	**0.95–71.1**	**8.88**	**1.00–78.5**
Tertile3	27	**4.17**	**1.37–12.7**	**3.90**	**1.25–12.1**	1.41	0.40–3.25	1.26	0.45–3.52	1.79	0.62–5.26	2.01	0.67–6.02	**3.00**	**1.04–8.64**	**2.90**	**0.99–8.52**	**8.60**	**0.97–76.4**	7.49	0.82–68.8
**Meal skipping eating pattern**																					
Tertile1	33	1	Ref.	1	Ref.	1	Ref.	1	Ref.	1	Ref.	1	Ref.	1	Ref.	1	Ref.	1	Ref.	1	Ref.
Tertile2	37	1.89	0.72–4.99	1.76	0.69–4.69	1.55	0.62–3.89	1.47	0.58–3.73	1.96	0.69–5.52	2.25	0.78–6.52	0.95	0.38–2.25	0.91	0.36–2.24	1.09	0.33–3.62	0.94	0.27–3.22
Tertile3	24	**4.85**	**4.85–15.1**	**4.62**	**1.45–14.6**	1.45	0.51–4.18	1.44	0.50–4.17	**3.06**	**0.99–9.41**	**3.87**	**1.18–12.6**	0.63	0.22–1.80	0.62	0.21–1.76	0.47	0.08–2.54	0.45	0.08–2.45
**Cultural and life style behavior eating pattern**																					
Tertile1	35	1	Ref.	1	Ref.	1	Ref.	1	Ref.	1	Ref.	1	Ref.	1	Ref.	1	Ref.	1	Ref.	1	Ref.
Tertile2	32	0.92	0.35–2.42	0.94	0.35–2.51	0.85	0.33–2.19	0.92	0.35–2.41	1.69	0.62–4.63	1.81	0.64–5.08	2.45	0.92–6.46	2.49	0.93–6.67	1.76	0.45–1.90	1.81	0.45–7.20
Tertile3	29	1.46	0.54–3.92	1.28	0.46–3.51	0.56	0.21–1.52	0.52	0.19–1.43	1.11	0.38–3.24	1.26	0.42–3.75	0.77	0.28–2.12	0.71	0.25–1.92	1.22	0.28–5.35	0.92	0.19–4.46

Ref, reference; OR, odds ratio; CI, confidence interval.

*Significant associations are presented in bold fonts (P < 0.05 or P < 0.01).

† Adjusted for background variables including age and body mass index.

Multiple logistic regression also showed that adolescents in high scores of “vitality” subscale of health related quality of life, had reduced likelihood of emotional disorders, conduct disorders and hyperactivity disorders; while adolescents in high scores of “mental health” components had reduced likelihood of emotional disorders and conduct problems (Table 4).

**Table 4:** Association between tertiles of components of quality of life scores and mental health problems among adolescents.

HRQoL components		Indicators of emotional disorders				Indicators of conduct problems				Indicators of hyperactivity disorders				Indicators of peer problems				Indicators of prosocial problems			
		Crude		Adjusted†		Crude		Adjusted †		Crude		Adjusted†		Crude		Adjusted†		Crude		Adjusted†	
	N	OR	95% CI	OR	95% CI	OR	95% CI	OR	95% CI	OR	95% CI	OR	95% CI	OR	95% CI	OR	95% CI	OR	95% CI	OR	95% CI
**Physical functioning**																					
Tertile1	29	1	Ref.	1	Ref.	1	Ref.	1	Ref.	1	Ref.	1	Ref.	1	Ref.	1	Ref.	1	Ref.	1	Ref.
Tertile2	37	0.98	0.37–2.60	0.98	0.37–2.60	0.95	0.36–2.48	0.95	0.36–2.48	0.58	0.21–1.67	0.58	0.20–1.68	0.61	0.23–1.59	0.61	0.23–1.58	0.34	0.08–1.52	0.35	0.08–1.52
Tertile3	21	0.70	0.23–2.17	0.64	0.21–2.21	1.75	0.56–5.40	1.67	0.54–5.22	1.36	0.44–4.24	1.36	0.43–4.39	1.12	0.37–3.36	1.11	0.34–3.18	0.65	0.15–2.97	0.47	0.08–2.52
**Role limitations due to physical difficulties**																					
Tertile1	36	1	Ref.	1	Ref.	1	Ref.	1	Ref.	1	Ref.	1	Ref.	1	Ref.	1	Ref.	1	Ref.	1	Ref.
Tertile2	28	**0.35**	**0.13–0.98**	0.32	0.11–0.93	0.59	0.22–1.59	0.54	0.19–1.46	0.91	0.32–2.57	0.97	0.34–2.78	0.99	0.37–2.63	1.01	0.37–2.72	0.72	0.21–2.47	0.61	0.16–2.27
Tertile3	31	0.46	0.17–1.22	0.45	0.17–1.22	0.95	0.36–2.49	0.98	0.37–2.58	0.64	0.23–1.82	0.61	0.21–1.76	0.37	0.84–0.98	0.34	0.12–0.93	0.12	0.01–1.03	0.11	0.01–0.99
**Role limitations due to emotional difficulties**																					
Tertile1	31	1	Ref.	1	Ref.	1	Ref.	1	Ref.	1	Ref.	1	Ref.	1	Ref.	1	Ref.	1	Ref.	1	Ref.
Tertile2	35	0.87	0.33–2.29	0.85	0.32–2.27	1.12	0.43–2.91	1.13	0.44–2.94	0.88	0.33–2.35	0.91	0.34–2.42	1	0.39–2.55	0.99	0.38–2.53	1	0.29–3.45	0.99	0.28–3.45
Tertile3	30	0.47	0.17–1.33	0.42	0.15–1.21	1.13	0.28–2.01	0.72	0.26–1.98	0.54	0.18–1.61	0.57	0.19–1.75	0.66	0.25–1.79	0.61	0.22–1.65	0.35	0.06–1.92	0.18	0.02–1.53
**Vitality subscale**																					
Tertile1	39	1	Ref.	1	Ref.	1	Ref.	1	Ref.	1	Ref.	1	Ref.	1	Ref.	1	Ref.	1	Ref.	1	Ref.
Tertile2	36	1.49	0.59–3.74	1.41	0.55–3.59	**0.45**	**0.18–1.13**	**0.38**	**0.14–0.99**	**0.30**	**0.11–0.82**	**0.32**	**0.11–0.83**	1.74	0.72–4.25	1.75	0.71–4.36	0.42	0.19–1.49	0.31	0.08–1.27
Tertile3	22	**0.27**	**0.08–0.91**	**0.26**	**0.07–0.86**	**0.17**	**0.05–0.55**	**0.15**	**0.04–0.49**	**0.31**	**0.11–0.97**	**0.30**	**0.10–0.98**	1.08	0.38–3.04	1.14	0.41–3.25	0.35	0.07–1.77	0.34	0.06–1.75
**Mental health**																					
Tertile1	30	1	Ref.	1	Ref.	1	Ref.	1	Ref.	1	Ref.	1	Ref.	1	Ref.	1	Ref.	1	Ref.	1	Ref.
Tertile2	53	**0.18**	**0.06–0.51**	**0.17**	**0.06–0.51**	**0.25**	**0.09–0.67**	**0.26**	**0.1–0.69**	0.46	0.18–1.14	0.45	0.18–1.14	0.67	0.28–1.59	0.68	0.28–1.65	1.66	0.48–5.81	2.12	0.55–8.26
Tertile3	13	**0.13**	**0.03–0.57**	**0.13**	**0.03–0.57**	**0.06**	**0.01–0.33**	**0.06**	**0.01–0.35**	0.22	0.04–1.14	0.21	0.03–1.10	0.52	0.14–3.25	0.52	0.13–1.96	0.62	0.06–6.81	0.79	0.07–8.45
**Social functioning**																					
Tertile1	36	1	Ref.	1	Ref.	1	Ref.	1	Ref.	1	Ref	1	Ref.	1	Ref.	1	Ref.	1	Ref.	1	Ref.
Tertile2	38	1.14	0.45–2.89	0.97	0.37–2.56	0.95	0.38–2.35	0.89	0.36–2.23	0.99	0.36–2.69	1.20	0.43–3.41	0.66	0.27–1.62	0.62	0.25–1.54	0.75	0.23–2.51	0.62	0.18–2.17
Tertile3	21	5.02	1.50–16.8	4.91	1.45–16.5	3.71	1.11–12.2	3.63	1.09–12.5	2.70	0.89–8.16	3.18	0.98–9.72	0.75	0.26–2.15	0.76	0.26–2.18	0.44	0.08–2.35	0.44	0.08–2.34
**Bodily pain**																					
Tertile1	33	1	Ref.	1	Ref.	1	Ref.	1	Ref.	1	Ref.	1	Ref.	1	Ref.	1	Ref.	1	Ref.	1	Ref.
Tertile2	30	1.16	0.42–3.23	1.22	0.44–3.44	1.76	0.65–4.81	1.76	0.65–4.87	2.04	0.64–6.51	2.15	0.65–7.05	2.47	0.92–6.62	2.59	0.96–7.02	1.97	0.43–9.03	2.06	0.44–9.59
Tertile3	34	3.20	1.18–8.71	3.35	1.18–9.48	3.83	1.40–10.4	3.72	1.32–10.4	3.60	1.19–10.8	4.02	1.26–12.7	1.26	0.48–3.31	1.30	0.48–3.52	2.48	0.59–10.5	2.32	0.52–10.4
**General health**																					
Tertile1	40	1	Ref.	1	Ref.	1	Ref.	1	Ref.	1	Ref.	1	Ref.	1	Ref.	1	Ref.	1	Ref.	1	Ref.
Tertile2	31	0.94	0.38–2.41	0.93	0.36–2.41	0.67	0.26–1.74	0.68	0.26–1.77	0.99	0.36–2.73	1.01	0.36–2.83	3.11	1.18–8.19	3.07	1.16–8.12	2.06	0.44–9.59	1.18	0.26–4.02
Tertile3	25	0.93	0.34–2.51	0.88	0.32–2.46	0.89	0.34–2.35	0.86	0.32–2.29	1.49	0.55–4.05	1.44	0.52–4.02	2.76	1.03–7.38	2.63	0.97–7.10	2.32	0.52–10.4	1.02	0.32–4.30

Ref, reference; OR, odds ratio; CI, confidence interval.

*Significant associations are presented in bold fonts (P < 0.05 or P < 0.01).

† Adjusted for background variables including age and body mass index.

## Discussion

In the current study a high prevalence of mental health problems in female adolescents was identified. Moreover, we found a significant relationship between behavioral eating patterns and mental health problems in female adolescents independent of age and BMI. Adolescents with higher adherence to “snacking and convenience”, “planning ahead” and “meal skipping” eating patterns were more likely to have indicators of emotional disorders. Additionally, adolescents in high scores of “low fat” eating pattern were less likely to have hyperactivity disorder.

In the current study, the prevalence of abnormal SDQ scores ranged between 3.73 to 25.23% in female adolescents. These percentages were comparable or even slightly higher compared with the previous reports of the prevalence of mental health problems in developing countries. Similar to our findings, in the study by Rabbani et al [49] the total prevalence of mental health problems evaluated by SDQ in female Iranian adolescents was 17.8% and the most prevalent mental health problem domain was conduct disorder (20.5%). In other study by Atilola et al [50] evaluating the prevalence of self-reported mental health problems using SDQ questionnaire among adolescents of five developing countries, 10.5% the prevalence of mental health problems was reported (range from 5.8 to 15%). The corresponding values of the prevalence of mental health problems in developed countries are lower. In the study by Oellingrath et al [2] the prevalence of combined borderline and abnormal SDQ scores among Norwegian adolescents ranged between 2–9%. Therefore residency and area of life are important determinants of mental health problems. In other study by Greally et al [51] the prevalence of abnormal and borderline SDQ scores among Irish adolescents were 8.7 and 15.3% respectively.

The major contributing factors in the higher prevalence of mental disorders in Iranian females are having multiple roles in the home and in the society, the effects of modernization and social media in defining thin ideals and make women with low self-esteem, more body image dissatisfaction and distortion, triggers the unhealthy eating patterns and eventually leads to mental health issues among this population [18, 29–31, 52].

The association between eating patterns and mental health can be explained by the association of eating low nutrient-dense foods with mental problems via the direct effect of diet on inflammatory parameters, immune system markers and biomarkers of oxidative stress responsible for mental issues like depression or anxiety and the effects of unhealthy food items like simple sugars and saturated fats on the proteins involved in the brain development like BDNF [14–18].

Several previous studies assessed the relationship between mental health problems and eating habits in adolescents. These studies mostly reported the association of unhealthy eating patterns with ADHD [9] depressive disorders and anxiety [7, 10]. Moreover, main meal skipping and high consumption of sugar-containing soft drinks have been associated with various mental health problems [8, 12, 13].

Most of the studies in this field have focused on diet or dietary patterns as a whole; while, in the current study we evaluated the association between eating behavioral patterns and mental health problems in female adolescents. In fact, there are different assessments of mental health problems and dietary patterns which make the comparison of results difficult; however several similarities could be addressed here. In the study by Ollingrath [2] adolescents with high scores of “varied Norwegian” eating pattern, defined as “nutrient-dense foods like fruits, vegetables, unrefined grains and fish combined with regular meals”, were less likely to have indicators of any psychiatric disorders. Moreover, similar to our results, high scores of “snacking” eating pattern was associated with higher indicators of mental health problems. “snacking and convenience”, “planning ahead” and “meal skipping” all are unhealthy eating behaviors that may lead to mental health problems; although these relationship might be bidirectional and mental health problems also may promote unhealthy eating patterns. In other word, eating behaviors might affect mental health by reinforcing healthy or unhealthy eating patterns (i.e., healthy eating patterns might makes people feel better psychologically, but it is also possible that unhealthy eating pattern might also make people feel better emotionally) [53].

In our study, eating low fat foods was associated with lower likelihood of hyperactivity disorder. The possible underlying mechanism is altered dopamine metabolism in high fat diet. In the Kaczmarczyk study, a high -fat diet was linked to childhood brain-based abnormalities, such as memory-dependent learning disabilities and ADHD by rapidly affecting dopamine metabolism in the brain, triggering anxious behaviors and learning deficiencies established in a juvenile mice model [54]. In their study high fat diet for one week impaired learning, memory and dopamine metabolism in hippocampus and hypothalamus. Similarly, several previous studies reported that “Western” and “fast food” dietary patterns were associated with hyperactivity disorder; one of the main features of these diets is their high fat content [55]. In other word, unhealthy food habits can trigger hyperactivity disorder as reported previously by Ollingrath [2]. In their study eating junk and convenient foods was associated with hyperactivity disorder. In addition, meal skipping was also positive predictor of hyperactivity disorder in our study. In fact, skipping meals specially breakfast and dinner is one of the main features of hyperactive individuals and disrupted eating behaviors are common among them [56, 57]. Skipping meals specially breakfast can alter mood, lead to mental distress, disrupt cognitive function and reduce academic performance in adolescents [13].

Eating patterns were associated with mental health problems in the current study, however, no significant difference was observed in comparison of BMI and nutrients intakes in different categorizes of mental health problems. This inconsistency might arise from this fact that relying on traditional single-nutrient approach in nutritional researches is misleading because of the complexity of human diets and high biochemical and nutritional interaction between various nutrients. Dietary pattern evaluates the effects of overall diet instead of single nutrients or food ingredients, and therefore, dietary patterns gives an extensive image of food and nutrient consumption and are more predictive of numerous chronic disease risks compared with single food ingredients or nutrients. In fact, the real eating behaviors of population could be achieved by measuring dietary patterns [58, 59].

In our study, high scores of health related quality of life were associated with lower experience of emotional, conduct and hyperactivity disorders in female adolescents. These findings were in consistent with previous studies reporting lower health related quality of life in patients with mental disorders [60–62].

Several limitations of the current study should also be addressed; first of all, the cross-sectional design of the study eliminates the possibility of identifying the causal relationship between adolescent’s eating patterns and mental health. Moreover, we did not assess several demographic factors possibly affect the mental status and eating patterns like the adolescents’ physical activity and socio-economic status of the family. Therefore it was not possible to adjust for the confounding effects of them. Moreover, most of our information obtained as self-reported data that does not take into account issues concerning honesty and accuracy of responses and therefore might be a source of recall bias and social desirability bias.

## Conclusion

In conclusion, our study showed a significant association between unhealthy eating patterns including ‘snacking and convenience’, ‘planning ahead’ and ‘meal skipping’ eating patterns with mental health problems. Our findings propose that unhealthy eating patterns are common predictors of mental health problems. The direction of the associations observed in the current study should be further evaluated by longitudinal or follow-up studies regarding the long-term effects of eating patterns on mental health on adolescents. Moreover, regarding the high prevalence of mental disorders among Iranian female adolescents and their relationship with unhealthy eating patterns, it is necessary to provide effective educational strategies to reduce the potential harm effects of unhealthy eating behaviors.

## Supporting information

S1 Dataset (SAV)

## Abbreviations

BMI: body mass index
SDQ: strengths and difficulties questionnaires
EBPQ: eating behavioral pattern questionnaire
FFQ: food-frequency questionnaire
HRQoL: health related quality of life
ADHD: attention deficit hyperactivity disorder
